# Flower Pots

**Published:** 1880-03

**Authors:** 


### *FLOWER POTS.*

Save the tin fruit cans, and convert them into tasteful flower pots in the following manner. With a can opener cut off any rough or projecting portions of the cover, leaving a narrow rim to project inward. With a pair of pliers, or a small hammer, bend this rim down. This gives firmness to the top of the can. Punch three or four small holes through the bottom of the can, Then paint it with varnish made of gum shellac dissolved in alcohol, and colored with lamp black and a little yellow ochre to give a dark brown color. The cans may be ornamented by pasting on them little medallion figures or pictures. They are handsomer than the ordinary flower pots, require less watering, and keep the plants free from all insects, owing to the presence of iron rust in the can. One of the prettiest arrangements for plants we have seen, was a window with two narrow shelves placed one above the other, on which were these home-made flower pots, containing heliotropes, geraniums, pinks, begonias, petunias, fuchias, and other plants, all as thrifty as if grown in a greenhouse. They should be showered once a fortnight with lukewarm water, using a whisp broom for the purpose, and watered sparingly every second day. On very cold nights newspapers may be placed between the window and the plants, to protect them from frost.

